# Immunological Characterization of HIV and SARS-CoV-2 Coinfected Young Individuals

**DOI:** 10.3390/cells10113187

**Published:** 2021-11-16

**Authors:** Claudia Vanetti, Daria Trabattoni, Marta Stracuzzi, Antonella Amendola, Clara Fappani, Valeria Rubinacci, Claudio Fenizia, Laura Gianolio, Mara Biasin, Anna Dighera, Irma Saulle, Elisabetta Tanzi, Gianvincenzo Zuccotti, Mario Clerici, Vania Giacomet

**Affiliations:** 1 Department of Biomedical and Clinical Sciences “L. Sacco”, University of Milan, 20157 Milan, Italy; daria.trabattoni@unimi.it (D.T.); claudio.fenizia@unimi.it (C.F.); mara.biasin@unimi.it (M.B.); irma.saulle@unimi.it (I.S.); 2 Pediatric Infectious Disease Unit, Department of Pediatrics, Luigi Sacco Hospital, University of Milan, 20157 Milan, Italy; valeria.rubinacci@unimi.it (V.R.); laura.gianolio@unimi.it (L.G.); anna.dighera@unimi.it (A.D.); vania.giacomet@unimi.it (V.G.); 3 Department of Health Sciences, University of Milan, 20133 Milan, Italy; antonella.amendola@unimi.it (A.A.); clara.fappani@unimi.it (C.F.); elisabetta.tanzi@unimi.it (E.T.); 4 Department of Pathophysiology and Transplantation, University of Milan, 20122 Milan, Italy; Mario.clerici@unimi.it; 5 Department of Pediatrics, V. Buzzi Children’s Hospital, University of Milan, 20154 Milan, Italy; gianvincenzo.zuccotti@unimi.it; 6 Pediatric Clinical Research Center “Romeo ed Enrica Invernizzi”, Department of Biomedical and Clinical Sciences “L. Sacco”, University of Milan, 20157 Milan, Italy; 7 IRCCS Fondazione Don Carlo Gnocchi, 20148 Milan, Italy

**Keywords:** HIV, COVID-19, SARS-CoV-2, IL-10, immune response, children, young adult, Calu-3

## Abstract

While the risk of SARS-CoV-2 infection and/or COVID-19 disease progression in the general population has been largely assessed, its impact on HIV-positive individuals remains unclear. We present clinical and immunological data collected in a cohort of HIV-infected young individuals during the first wave of COVID-19 pandemic. SARS-CoV-2 RNA, virus-specific antibodies, as well as the expression of factors involved in the anti-viral immune response were analyzed. Moreover, we set up an in vitro coinfection assay to study the mechanisms correlated to the coinfection process. Our results did not show any increased risk of severe COVID-19 in HIV-positive young individuals. In those subjects who contracted SARS-CoV-2 infection, an increase in IL-10 expression and production was observed. Furthermore, in the in vitro coinfection assay, we revealed a reduction in SARS-CoV-2 replication associated to an upregulation of IL-10. We speculate that IL-10 could play a crucial role in the course of SARS-CoV-2 infection in HIV-positive individuals. These results might help defining clinical management of HIV/SARS-CoV-2 co-infected young individuals, or putative indications for vaccination schedules in this population.

## 1. Introduction

SARS-CoV-2-specific immune responses in young patients are still scarcely described. Although it is known that COVID-19 severity grows within age groups, the paucity of data present in literature do not allow one to fully understand the clinical and immunological basis of this phenomenon [1]. Very limited results, in particular, are available in HIV-infected young patients, in whom several assumptions have been made based on their immunocompromised status. On one hand, it was suggested that immunosuppression and a high viral load, which are common risk factors for opportunistic viral infections, could facilitate SARS-CoV-2 infection in this vulnerable subgroup [2,3]. Conversely, an impaired immune system was hypothesized to hamper the cytokine storm that is associated with severe COVID-19 [4]. Different theories have also been presented to understand the possible role played by antiretrovirals (ARVs) in SARS-CoV-2 infection. Thus, whereas long-term ARV treatment could result in those same chronic comorbidities (including cardiovascular diseases, diabetes, dyslipidemia, renal impairment, and metabolic alteration) that are associated with a more severe COVID-19 course, antivirals were tentatively used in the therapy of COVID-19 [4]. Available results indicate that HIV-positive adult patients affected by COVID-19 exhibit clinical features and a disease course comparable to the one observed in the general population [5,6,7]. However, immunological and clinical data on HIV-infected young patients are still sorely missing [7]. 

In the attempt to fill this gap, we evaluated viro-immunological responses in a cohort of HIV-infected young subjects who contracted SARS-CoV-2 infection (H+/S+) during the first wave of the COVID-19 pandemic. Results were compared to those obtained in a group of HIV-positive, SARS-CoV-2-negative individuals (H+/S-) and in a cohort of SARS-CoV-2-positive, HIV-negative age-matched patients (H−/S+). Furthermore, we devised an in vitro model of coinfection to unravel the potential mechanisms associated with the HIV/SARS-CoV-2 interplay.

## 2. Materials and Methods

### 2.1. Enrollment and Clinical Evaluation

Between April and September 2020, we enrolled 85 ART-treated HIV-infected young patients followed up at the Unit of Pediatric Infectious Diseases, Sacco Hospital in Milan. Biological samples were collected during follow up visits. Patients with a positive result on high throughput sequencing, real-time reverse-transcriptase–polymerase-chain-reaction (RT-PCR) assay of sputum specimens, or positive results on serum anti-SARS-CoV-2-specific IgG were considered as acute or previous SARS-CoV-2 infected subjects, respectively. Four out of 85 patients who resulted SARS-CoV-2-infected were compared to 6 H+/S− and then to 7 HIV−/S+.

Data regarding the history of recent COVID-19 exposure, clinical symptoms, or signs were extracted from medical record. The prospective study was approved by the Ethical Committees (protocol number 0034645, approved on 8 November 2020).

HIV-RNA viral load (VL) detection and CD4+ T cell count were performed in all HIV-infected patients at the time of enrollment.

Furthermore, 10 HIV-seronegative, SARS-CoV-2-negative, and not vaccinated young volunteers (H−/S−), were enrolled for in vitro coinfection experiments.

### 2.2. SARS-CoV-2 Genome Quantification

All participants underwent SARS-CoV-2 testing after study recruitment. After collection of sputum, recently reported to be as sensitive as swab samples [8], specimens were immediately processed. A Maxwell^®^ RSC Viral Total Nucleic Acid Purification Kit (Promega, Fitchburg, WI, USA) was used to extract SARS-CoV-2 RNA using the Maxwell^®^ RSC Instrument (Promega, Fitchburg, WI, USA). Viral RNA was quantified as previously described [9]. Briefly, a single-step RT PCR-time PCR (GoTaq^®^ 1-Step RT-qPCR) (Promega, Fitchburg, WI, USA) and the 2019-nCoV CDC qPCR Probe Assay emergency kit (IDT, Coralville, IA, USA) were used on a CFX96 instrument (Bio-Rad, Hercules, CA, USA). Viral copy number quantification was performed by generating a standard curve from the quantified 2019-nCoV_N-positive Plasmid Control (IDT, Coralville, IA, USA). A cycle threshold (Ct) value of <40 was considered positive, based on CDC guidelines.

### 2.3. SARS-CoV-2 Specific Antibodies

Serum was tested for anti-SARS-CoV-2-specific IgG using the semi-quantitative Anti-SARS-CoV-2 ELISA (Euroimmun, Lübeck, Germany) test, according to the manufacturer’s instructions. Specifically, 10 µL of each serum were diluted 1:101 in the sample buffer, and 100 µL of diluted serum were incubated into individual microplate wells coated with an S1 domain of the spike protein of SARS-CoV-2 expressed recombinantly in the human cell line HEK 293. Results were assessed semi-quantitatively by a ratio (Optical Density sample/Optical Density calibrator) and interpreted as follows: <0.8 negative, ≥0.8 to <1.1 borderline, ≥1.1 positive.

### 2.4. Quantigene Plex Gene Expression Analysis

To study the SARS-CoV-2 specific immune response, 1 × 10^5^ peripheral blood mononuclear cells (PBMCs), isolated from subjects enrolled as previously described [9], were stimulated with SARS-CoV-2 antigens (500 ng/mL nucleocapsid (N) and spike (S), Novatein Biosciences, Woburn, MA, USA) for 10 h. After 10 h of stimulation, PBMCs were harvested and gene expression analyses were performed by QuantiGene Plex assay (Thermo Scientific, Waltham, MA, USA), a technique allowing the simultaneous measurement of 70 selected genes involved in the antiviral/immune response in a single well of a 96-well plate.

### 2.5. Multiplex Analysis

A 27-cytokine multiplex assay was performed on patients’ plasma using magnetic bead immunoassays (Bio-rad, Hercules, CA, USA) and Bioplex 200 Systems (Bio-rad, Hercules, CA, USA). Some of the targets resulted to be over-range and arbitrary value of 4000 pg/mL was assigned, while 0.1 pg/mL was attributed to values below the limit of detection.

### 2.6. In Vitro Cell Culture, Co-Culture, and Infection Assay

Calu-3 (HTB-55™, human epithelial cells from lung adenocarcinoma) cells were purchased from American Type Culture Collection (ATCC^®^, Manassas, VA, USA). Calu-3 cells were grown in DMEM high glucose, 2 mM Glutamax, PenStrep, 10% FBS, and 1% NEAA.

**Scheme 1:**
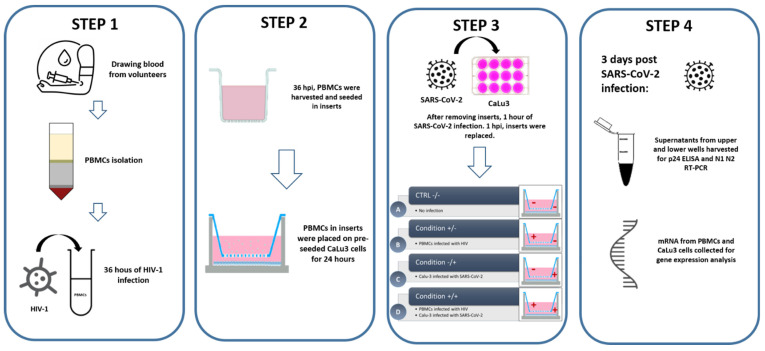
Experimental design of the in vitro coinfection assay.

For SARS-CoV-2/HIV-1 coinfection (Scheme 1), 1.25 × 10^5^ Calu-3 cells were cultured in 1 mL 2% FBS medium in a 12 well plate with 0.4 µm pore polycarbonate membrane inserts (Costar, Corning Incorporated, Corning, NY, USA). The same day, 2 × 10^6^ PBMCs, isolated from HIV-negative, SARS-CoV-2-negative volunteers, were cultured in 1 mL RPMI 20% FBS medium in FACS tubes and challenged with 1 ng/1 × 10^6^ cells of p24 HIV-1_Bal_. At 36 h post infection (hpi), PBMCs were harvested, washed in pre-warmed PBS, and 1 × 10^6^ cells were cultured in 1 mL RPMI 2% FBS medium with 15 ng/mL of IL-2 in the upper chamber of the insert membranes containing 0.4 μm pore filters placed on the pre-seeded Calu-3 cells. After 24 h, Calu-3 cells were challenged with SARS-CoV-2 (Virus Human 2019-nCoV strain 2019-nCoV/Italy-INMI1, Rome, Italy), propagated in Caco2 cell line, at a multiplicity of infection (MOI) of 0.015 for one hour. Calu-3 cells were than thoroughly washed three times with pre-warmed PBS and refilled with proper growth medium (DMEM 10% FBS).

At 96 hpi (6 days after HIV-1 infection), both PBMCs and Calu-3 cell lines were lysed for RNA extraction, whereas supernatants were collected from upper wells for p24 antigen ELISA (Cell Biolabs, San Diego, CA, USA) and from lower wells to evaluate SARS-CoV-2 replication rate (Scheme 1) using the viral RNA extraction method and the SARS-CoV-2-RNA detection protocol previously described [9] and reported in Section 2.2. An enzyme-linked immunosorbent assay (ELISA) kit was purchased from R & D Systems Inc. (Minneapolis, MN, USA) to quantify interleukin-10 (IL-10) in supernatants from co-cultures. All the experiments with HIV-1 and SARS-CoV-2 virus were performed in the BSL3 facility.

### 2.7. Gene Expression Analyses by RT-QPCR

RNA was extracted from PBMCs and Calu-3 cell lines by using the acid guanidium thiocyanate–phenol–chloroform method. RNA was dissolved in RNase-free water, quantified by the Nanodrop 2000 Instrument (Thermo Scientific) and purified from genomic DNA with RNase-free DNase (RQ1 DNase; Promega). One microgram of RNA was reverse transcribed into first-strand cDNA as previously described [10], with a 20 μL final volume containing 1 μM random hexanucleotide primers, 1 μM oligo dT and 200 U Moloney murine leukemia virus reverse transcriptase (Promega, Fitchburg, WI, USA). cDNA quantification for interleukin (IL) 6, IL8, IL10, IFNG, TGFβ1, STAT3 was performed on PBMCs as well as on Calu-3 cells by real-time PCR (CFX96 connect, Bio-Rad, Hercules, CA, USA). Reactions were performed using a SYBR Green PCR mix (Promega, Fitchburg, WI, USA) and amplified according to the following thermal profile: initial denaturation (95 °C, 15 min) followed by 40 cycles of 15 s at 95 °C (denaturation) and 1 min at 60 °C (annealing) and 20 s at 72 °C (extension). Furthermore, a melting curve analysis was assessed for amplicon characterization. A Ct value of 40 or higher means no amplification and this value was not included in the calculations. Results are shown as the media of the relative expression units to the glyceraldehyde-3-phosphate dehydrogenase (GAPDH) housekeeping gene calculated by the 2^−ΔΔCt^ equation. Primers sequences were the following: GAPDH primers: forward CGGATTTGGTCGTATTGGG, reverse GCTTCCCGTTCTCAGCCTTG; IL8 primers: forward: TGGACCCCAAGGAAAACTGG, reverse: GCAACCCTACAACAGACCCACA; IL10 primers: forward: CTCCACGGCCTTGCTCTTGT, reverse: TCAAGGCGCATGTGAACTCC; IFNG primers: forward GGCGACAGTTCAGCCATCAC, reverse TGTGGAGACCATCAAGGAAGACA. For the detection of IL6, TGFβ1 and STAT3, primers were purchased already optimized (PrimePCR, Bio-Rad, Segrate, Italy).

### 2.8. Statistical Analysis

The Student’s *t*-test, the χ^2^ method, and Fisher’s exact test were done when appropriate for statistical analysis to compare continuous and categorical variables. One-way ANOVA was applied for parametric multiple comparison. A *p*-value < 0.05 was chosen as the cutoff for significance. Data were analyzed with StataMed (version 20.0) and GraphPad Prism 9.

## 3. Results

### 3.1. Clinical Aspects

Eighty-five HIV-infected young patients (94% of them were HIV-vertically infected; mean age 22.3 years; range 1–35 years) were enrolled in the study (Table 1). During the lockdown (April–May 2020), 14 patients declared not self-isolating at home because of work-related reasons. From May to September 2020 all patients were free to circulate in community. Patients were distinguished in two classes of severity according to clinical complexity/aspect: we considered patients as pauci-symptomatic or with moderate disease if they had upper or lower respiratory tract infection; otherwise, they are classified as severe/critical if they required oxygen supplementation or if they experienced multiorgan failure.

HIV-RNA was undetectable (<20 cp/mL) in 79 of 85 patients (93%); in the others the mean was 62.8 cp/mL (range < 20 detectable–160 cp/mL). The CD4 T cells count was >350 mm^3^ in 81 subjects (95%, average was 772.4 mm^3^, range was 363–2554 mm^3^), while in four patients the range was 230–345 mm^3^. All patients were full virological (HIV RNA < 20 CP) and immunological (CD4 > 350 mm^3^) responders and were receiving ARV treatments based on integrase inhibitors (40), protease inhibitors (31), nucleoside reverse transcriptase inhibitors (1), and non-nucleoside reverse transcriptase inhibitors (13).

Of these 85 patients, 10 (11%) had comorbidities as follows: three nephropathies, two neurological disorders, three HIV-related spastic paralysis and two hepatitis; moreover, coinfections were present in five out 85 (6%) patients (two were HBV-positive, three had been HCV-positive but had cleared the virus through antiviral therapy before the study’s beginning).

Among HIV-infected patients, SARS-CoV-2 RNA was detected in only one patient (sputum specimen) who tested negative for SARS-CoV-2 IgG. SARS-CoV-2 infection elicited IgG production in three other HIV-infected patients, who, however, were negative for SARS-CoV-2 RNA detection on sputum samples. Two of these three SARS-CoV-2-seropositive patients continued working during lockdown, one of the three was probably infected by a household contact. All H+/S+ patients were treated with a PI-based regimen, their HIV-RNA was undetectable, and CD4+ T cells were >350 mm^3^. The HIV-infected patient, whose sputum resulted positive to SARS-CoV-2 RNA, was in treatment with a PI-based regime and the CD4+ T cell average was 782 mm^3^. Inflammatory markers, as well as markers of liver and renal function and of coagulation, were within normal range throughout the study period in all the patients we examined. None of them were hospitalized for coronavirus-related symptoms; none received SARS-CoV-2 related antiviral or antibiotic treatment; none of them had undergone an antiretroviral treatment switch in the last 12 months. 

We compared the four H+/S+ patients to six out of 85 H+/S− subjects. Next, we compared H+/S+ subjects to a group of seven H−/S+ young subjects (mean age 22.8 years; range 10.8–40 years) (Table 1). Within the group of H−/S+ individuals, no comorbidities were observed. RT-PCR for SARS-CoV-2 on nasopharyngeal swab was positive in all cases; they all were undergoing house confinement and they all contracted infection from a household contact. All patients were pauci-symptomatic or showed mild clinical manifestations. 

### 3.2. H+/+ Subjects Reported Higher Production and mRNA Expression of IL-10 When Compared to H+/S− Individuals

**Figure 1:**
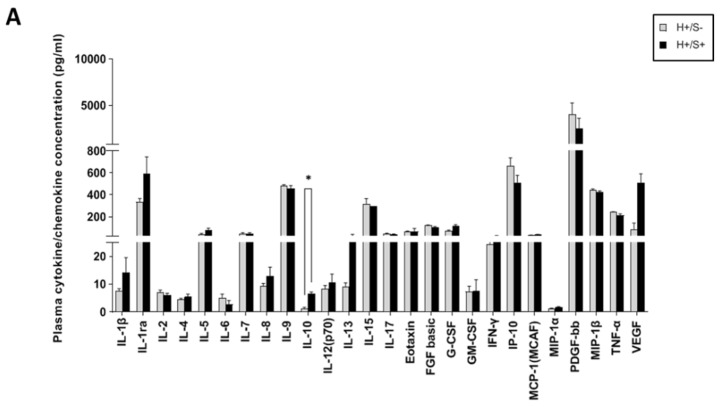

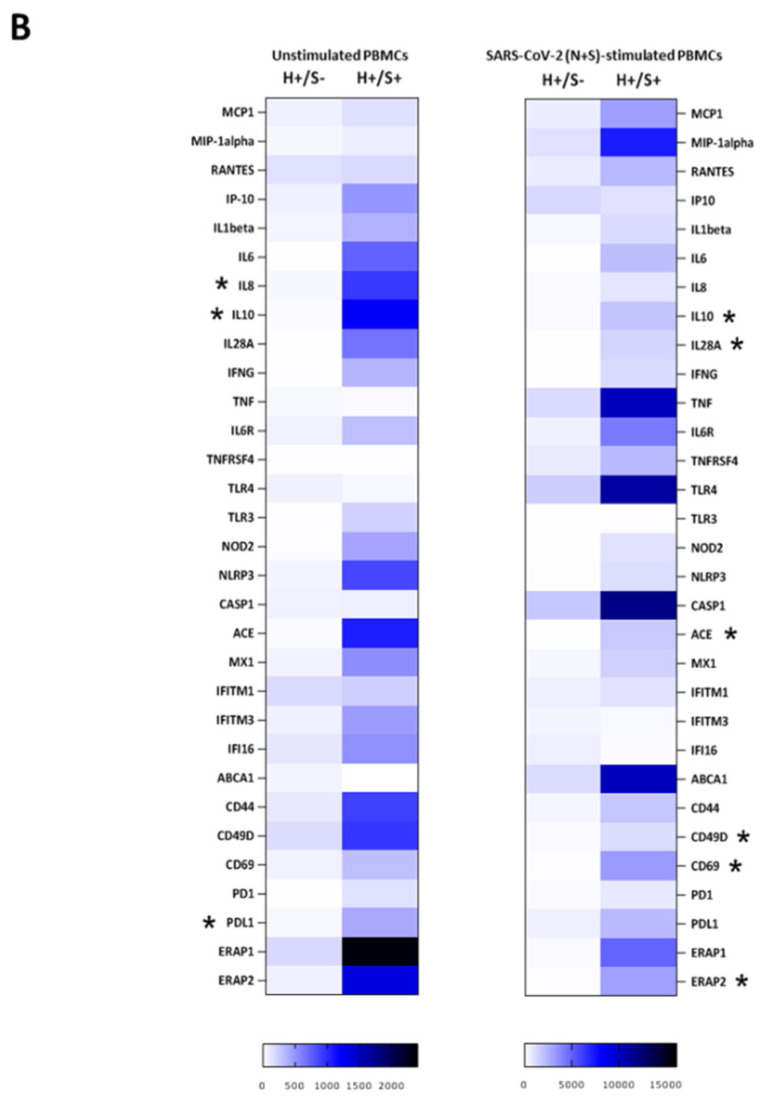
Immunological responses in H+/S− vs. H+/S+ individuals. (**A**) Plasma cytokine/chemokine concentrations (pg/mL) in H+/S− (grey bars) and H+/S+ (black bars) patients; (**B**) mRNA expression of genes involved in the antimicrobial immune response in unstimulated (left panel) and SARS-CoV-2 (N+S)-stimulated (right panel) PBMCs isolated from H+/S− and H+/S+ individuals; gene expression (mean values) is shown as a color scale from white to blue/black (Heatmap). Results are indicated as mean ± SEM values and statistically significant differences (*p* < 0.05) are indicated; * *p* < 0.05.

No differences were found in plasma cytokine and chemokine concentrations in H+/S+ coinfected subjects compared to H+/S− individuals (Figure 1A). The expression of genes involved in the antiviral immune responses was upregulated in H+/S+ coinfected subjects, with a significant higher expression of mRNA specific for IL8, IL10, and PDL1 (*p* < 0.05) in basal condition and IL10, IL28A, ACE, CD49D, CD69, and ERAP2 (*p* < 0.05) upon SARS-CoV-2 specific stimulation (Figure 1B). Finally, IL-10 production and mRNA expression were significantly increased in H+/S+ compared to H+/S− individuals (*p* < 0.05) (Figure 1A,B).

### 3.3. H+/S+ Patients Showed a Peculiar Inflammatory Profile When Compared to H−/S+ Individuals

**Figure 2:**
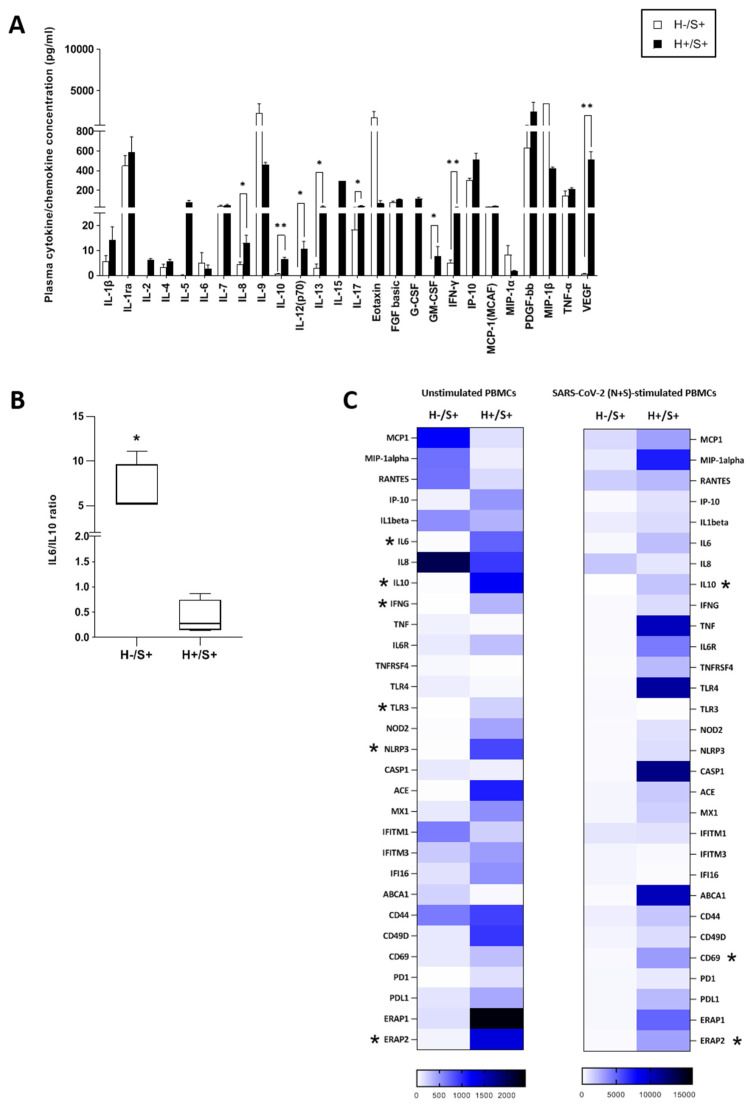
Immunological responses in H−/S+ vs H+/S+ individuals. (**A**) Plasma cytokine/chemokine concentrations (pg/mL) in H−/S+ (white bars) and H+/S+ (black bars) patients; (**B**) Plasma IL6/IL10 ratio in H−/S+ and H+/S+ patients; (**C**) mRNA expression of genes involved in the antimicrobial immune response analyzed on unstimulated (left panel) and SARS-CoV-2 (N+S)-stimulated (right panel) PBMCs isolated from H−/S+ and H+/S+ individuals; gene expression (mean values) is shown as a color scale from white to blue/black (Heatmap). Results are indicated as mean ± SEM values and statistically significant differences (*p* < 0.05) are indicated; * *p* < 0.05, ** *p* < 0.01.

A widespread hyper-activation characterized H+/S+ when they were compared to H−/S+ infected individuals, as the concentration of several cytokines and chemokines was significantly upregulated in the plasma of coinfected patients (IL-8, IL-12, IL-13, IL-17, GM-and CSF, *p* < 0.05; IL-10, IFNγ, and VEGF, *p* < 0.01) (Figure 2A). These results were confirmed by gene expression analysis in basal condition (IL6, IL10, IFNG, TLR3, NLRP3, and ERAP2; *p* < 0.05) and upon SARS-CoV-2 specific stimulation (IL10, CD69 and ERAP2; *p* < 0.05) (Figure 2C). Notably, the IL-6/IL-10 ratio in plasma was significantly reduced in H+/S+ compared to H−/S+ patients (*p* < 0.05) (Figure 2B). 

In line with the results of the comparison between HIV-positive individuals, HIV/SARS-CoV-2 coinfection was associated with higher IL10 expression than in HIV-negative SARS-CoV-2-positive individuals. 

### 3.4. In Vitro HIV/SARS-CoV-2 Coinfection Assays Confirmed the Upregulation of IL-10 

**Figure 3:**
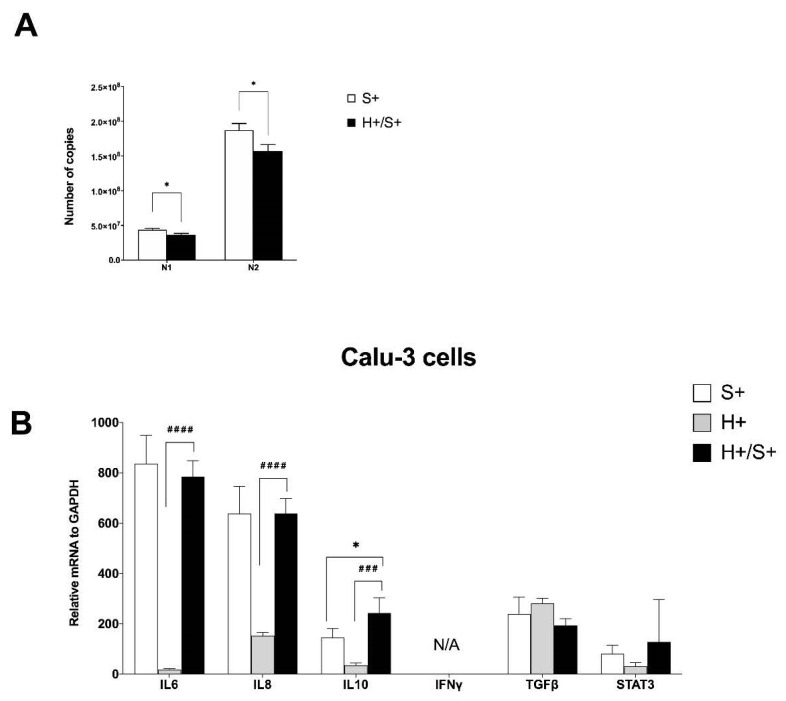

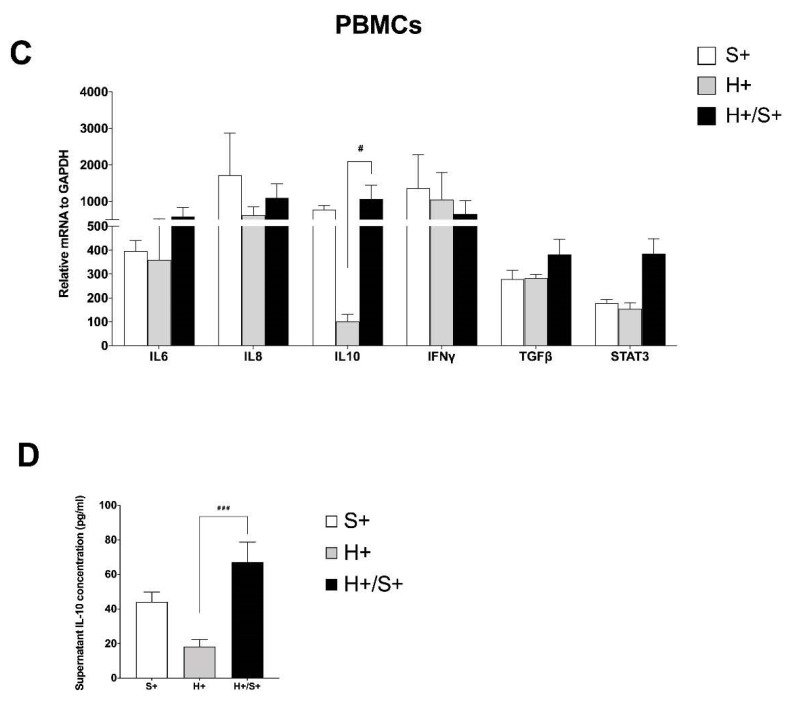
IL-10 expression and production are increased in in vitro coinfection. (**A**) Viral load expressed as copy number of N1 and N2 genes in SARS-CoV-2 infected only Calu-3 cells (white bar) and SARS-CoV-2-infected Calu-3 cells exposed to HIV-infected PBMCs (black bar). (**B**) mRNA expression of IL6, IL8, IL10, IFNG, TGFβ1, and STAT3, assessed by RT-Real-Time PCR, on Calu-3 from different conditions: Calu-3 infected only with SARS-CoV-2 (S+, white bar), not-infected Calu-3 cells exposed to HIV-infected PBMCs (H+, grey bar), and Calu-3 SARS-CoV-2-infected exposed to HIV-infected PBMCs (H+/S+, black bar); (**C**) mRNA expression of IL6, IL8, IL10, IFNG, TGFβ1, and STAT3, assessed by RT-Real-Time PCR, on PBMCs from different conditions: not-infected PBMCs exposed to SARS-CoV-2-infected Calu-3 (S+, white bar), HIV-infected only PBMCs (H+, grey bar), and HIV-infected PBMCs exposed to SARS-CoV-2-infected Calu-3 cells (H+/S+, black bar). Results are shown as the media of the relative expression units to the glyceraldehyde-3-phosphate dehydrogenase (GAPDH) as reference gene calculated by the 2^−ΔΔCt^ equation. (**D**) Supernatant IL-10 concentrations (pg/mL) from in vitro co-cultures. Results are expressed as mean ± SEM and statistically significant differences (*p* < 0.05) are indicated; * *p* < 0.05; **#** *p* < 0.05; ### *p* < 0.001; #### *p* < 0.0001.

To explore in details the mechanisms underlying the interaction between HIV and SARS-CoV-2 coinfection, we set up a coinfection assay with HIV-1 and SARS-CoV-2 using PBMCs from healthy (HIV- and SARS-CoV-2-negative) individuals that were co-cultured with Calu-3 lung epithelial cell lines. PBMCs infected with HIV-1 ex vivo, or uninfected cells, were transferred onto the membrane of a transwell insert pre-seeded with Calu-3 cells that were then exposed to SARS-CoV-2, or medium, (Scheme 1). SARS-CoV-2 replication in Calu-3 cells from the co-cultures showed a significant reduction when Calu-3 cells were exposed to HIV-infected PBMCs in comparison to uninfected PBMCs (Figure 3A). 

Focusing on Calu-3 cells from the co-cultures (Figure 3B), an increased gene expression of IL6, IL8, and IL10 was observed in Calu-3 cells in the coinfected condition compared to uninfected Calu-3 exposed to HIV pre-infected PBMCs. The levels of IL10, but not IL6 and IL8, in Calu-3 cells from coinfected cultures were also higher compared with SARS-CoV-2-infected Calu-3 exposed to uninfected PMBCs. These results were partly mirrored by gene expression analysis in PBMCs from the same co-cultures (Figure 3C). IL10 was the only cytokine to be significantly upregulated in PBMCs from coinfected cultures compared to infection with solely HIV-1, whereas no difference was observed between PBMCs from a coinfected condition and uninfected PBMCs exposed to SARS-CoV-2-infected Calu-3. These results suggest that the effects of HIV on SARS-CoV-2 occurred mainly by IL-10 action. A trending increase in STAT3 was observed in the coinfected condition compared to the other two groups (Figure 3C).

In the culture supernatants, we observed an increased IL-10 production in the coinfected condition, significantly higher when compared to the condition of HIV-infected PBMCs (*p* < 0.001) (Figure 3D). 

## 4. Discussion

In our center, we are following almost 100 HIV-seropositive young patients mostly with vertically-acquired infections. As these are fragile patients, we were concerned about a possible increased risk of severe COVID-19. For this reason, we started monitoring them with an active surveillance, ensuring our programmed controls and maintaining the proper safety measures. 

Our primary hypothesis was that HIV-infected patients could experience a more severe disease upon SARS-CoV-2 infection than immunocompetent hosts. 

However, our results show no increased risk or increased clinical severity of COVID-19 in HIV-positive young individuals, confirming previously published data [4,11,12,13] that showed that SARS-CoV-2 infection prevalence in HIV-infected young patients is comparable or lower than HIV-uninfected individuals belonging to the same age group. In a retrospective study comparing 21 people living with HIV (PWH) to 42 matched HIV-negative controls hospitalized at NYU Langone Health with COVID-19 between 2 March 2020 and 23 April 2020, a similar burden of comorbidities and similar admission laboratory values were seen between the two cohorts [14]. 

Despite a higher systemic immune activation in comparison to H−/S+ patients, as revealed by plasma cytokine analysis, our results show no increased risk or increased clinical severity of COVID-19 in HIV-positive young individuals, which is in line with previous studies [11]. 

To study in detail the interaction between the two viruses, we established an in vitro co-culture model involving SARS-CoV-2-infection of Calu-3 cells with PBMCs pre-infected with HIV-1 to investigate their cross-talk during the coinfection process. In this model, we observed, first of all, a reduction in SARS-CoV-2 replication in the supernatants obtain from Calu-3 cells exposed to HIV pre-infected PBMCs. Different examples of virus–virus negative interference have been reported in the literature. Several coinfections have the potential to inhibit other coronaviruses replications, such as human coronavirus NL63 (hCoV-NL63) [15], and because of the existing infection with HIV-1 interfering with the replication of the hCoV in the same host, it was suggested that the viral load of the hCoV remains low [16]. Next, we study gene expression in Calu-3 cells and PBMCs from every conditions of the co-culture. An upregulation in IL10 expression in the presence of HIV and SARS-CoV-2 infections in both Calu-3 cells and PBMCs, in comparison to the other conditions of single infection, was reported. This result was also confirmed in the analysis of cytokine concentration in supernatants collected from the co-cultures, where once again, higher levels of IL-10 were observed in the coinfected condition. In the attempt to study the mechanisms associated to the upregulation of IL-10 in the HIV/SARS-CoV-2-coinfected condition, we focused on the role of the Signal transducer and activator of transcription (STAT)3, known to be essential for all known functions of IL-10 [17]. IL-10 exerts its effects by binding to its cognate receptor (IL-10R). IL-10 binding to IL-10R activates the IL-10/JAK1/STAT3 cascade, where phosphorylated STAT3 homodimers translocate to the nucleus to activate the expression of target genes [18]. It has therefore been well demonstrated that both IL-10 and STAT3 are essential for the anti-inflammatory response. In our HIV/SARS-CoV-2 co-culture in vitro model we observed that the upregulation of IL10 is indeed associated to higher levels of STAT3. Notably, the higher IL10 expression that was observed in the coinfected condition was associated also with a slight upregulation of TGFβ, best known for its regulatory activity [19]. Together, IL-10 and TGF-β orchestrate antiviral immune responses turning off. The dampening in SARS-CoV-2 replication observed in the HIV/SARS-CoV-2-coinfected condition could confirm the possible protective role of this anti-inflammatory cytokine in HIV-positive patients. 

The role of IL-10 in SARS-CoV-2 infection has been recently thoroughly debated [20]. Some studies reported an association between early high IL-10 levels and poor clinical outcomes in severe COVID-19 cases [21]. Other results [22] showed that the ratio of IL-6 to IL-10 outperformed IL-6 alone in predicting clinical outcome, suggesting the importance of the balance between pro- and anti-inflammatory cytokines in determining the severity of COVID-19 infection. An increase in IL-10 production in HIV-infected individuals who contracted SARS-CoV-2 infection has been described in other studies [23]. SARS-CoV-2 itself activates pro-inflammatory genes in lung epithelial cells [24,25] and in PBMCs [24]. In the HIV-infected individuals enrolled in our study IL-10 production and expression were significantly increased in H+/S+ compared to all other groups, similarly to data obtained in the in vitro coinfection assay. We speculate that the increased expression of IL-10 observed in the HIV-positive population could play a role in the course of SARS-CoV-2 infection, as IL-10 has emerged as a key regulator of immune responses against viral infections [26]. 

Our study has some limitations. First, the relatively small sample size could lead to higher variability. Second, all the HIV-infected individuals who were enrolled in the study are immunological and virological responders, presenting a good compliance to ARV treatments. This may minimize the functional differences that we observed when H+/S+ and H−/S+ individuals were compared. Furthermore, we cannot exclude that H+/S+ patients could have benefited from ARV, as therapy might play a role in limiting the risk of severe COVID-19 clinical manifestations. Notably, recent results stemming from a comparison of HIV-infected patients who became SARS-CoV-2-infected and were or were not undergoing ARV indicated that HIV infection in the absence of ARV is a dangerous comorbidity [27]. However, this study also has the strength to provide insights into the dynamics of immune response to SARS-CoV-2 infection in HIV-positive individuals. 

Further investigations on HIV+ SARS-CoV-2-infected young patients are needed. We also need more evidence based on clinical and immunological findings in order to implement better therapeutic strategies. This could help the medical world not only to manage these fragile patients but also perhaps to better understand the pathogenesis of COVID-19 in the general population.

## Figures and Tables

**Table 1:** Demographic and clinical characteristics of enrolled patients.

	H+ (85)	H+/S+ (4)	H−/S+ (7)
Median age (range) in years	22.3 (1–35)	22.5 (19–30)	22.8 (10.8–40)
Male	35	1	4
Comorbidities	10 (11%)	0	0
Coinfections	5 (4.25%)	0	0
CD4 > 350 mm^3^	81 (95.3%)	4 (100%)	-
HIV RNA < 20 CP	79 (93%)	4 (100%)	-
Integrase inhibitors	40 (47%)	0	-
Protease inhibitors	31 (36.5%)	4 (100%)	-
Nucleoside reverse transcriptase Inhibitors	1 (1.17%)	0	-
Non-nucleoside reverse transcriptase inhibitors	13 (11%)	0	-
Positive RT-PCR on sputum	1 (1.17%)	1	7
IgG Anti-SARS-CoV-2	3 (3.53%)	3	-
Asymptomatic	85 (100%)	4 (100%)	0
Paucisyntomatic/moderate	0	0	7 (100%)
Severe/critical	0	0	0

## Data Availability

The data that support the findings of this study are available on request from the corresponding author, M.S. The data are not publicly available due to their containing information that could compromise the privacy of research participants.
